# A systematic literature review of ride-sharing platforms, user factors and barriers

**DOI:** 10.1186/s12544-021-00522-1

**Published:** 2021-12-07

**Authors:** Lambros Mitropoulos, Annie Kortsari, Georgia Ayfantopoulou

**Affiliations:** Centre for Research and Technology Hellas, Hellenic Institute of Transport, 52 Egialias Street, 15125 Marousi, Greece

**Keywords:** Ride-sharing, User factors, Platform, Carpooling

## Abstract

**Aim:**

Ride-sharing is an innovative on-demand transport service that aims to promote sustainable transport, reduce car utilization, increase vehicle occupancy and public transport ridership. By reviewing ride-sharing studies around the world, this paper aims to map major aspects of ride-sharing, including online platforms, user factors and barriers that affect ride-sharing services, and extract useful insights regarding their successful implementation.

**Method:**

A systematic literature review is conducted on scientific publications in English language. Articles are eligible if they report a study on user factors affecting ride-sharing use and/or barriers preventing ride-sharing implementation; ride-sharing online platforms in these articles are also recorded and are further explored through their official websites. A database is built that organizes articles per author, year and location, summarizes online platform attributes, and groups user factors associated with the likelihood to ride-share.

**Findings:**

The review shows that the term “ride-sharing” is used in the literature for both profit and non-profit ride-sharing services. In total, twenty-nine ride-sharing online platforms are recorded and analyzed according to specific characteristics. Sixteen user factors related to the likelihood to ride-share are recorded and grouped into sociodemographic, location and system factors. While location and system factors are found to follow a pattern among studies, mixed findings are recorded on the relationship between sociodemographic factors and ride-sharing. Factors that may hinder the development of ride-sharing systems are grouped into economic, technological, business, behavioral and regulatory barriers.

**Conclusion:**

Opportunities exist to improve the quality of existing ride-sharing services and plan successful new ones. Future research efforts should focus towards studying ride-sharing users' trip purpose (i.e., work, university, shopping, etc.), investigating factors associated to ride-sharing before and after implementation of the service, and perform cross-case studies between cities and countries of the same continent to compare findings.

## Introduction

Ride-sharing aims to minimize negative impacts related to emissions, reduce travelling costs and congestion [20, 40], and increase passenger vehicle occupancy and public transit ridership. During the last decade, innovative mobility solutions were introduced, including on-demand mobility services and Mobility as a Service (MaaS), that focused on daily travel needs to promote sustainable transport [20].

The literature uses the term “ride-sharing” to describe various mobility sharing concepts. Ride-sharing refers to the common use of a motor vehicle by a driver and one or several passengers, in order to share the costs (non-profit) or to compensate the driver (i.e., paid service) using billing information provided by the participants (for profit). In this study the term is used to describe the common use of a motor vehicle for cost compensation, in the context of a ride, that the driver performs for its own account (referred also as Carpooling); thus, it is not intended to result in any financial gain [20].

Practical experience shows that ride-sharing trips are usually pre-arranged through matching applications, that allow drivers and passengers to find potential rides. They often include community-based trust mechanisms, such as user-ratings and provide links to social networks to allow prospective sharers to check each other. Ride-sharing has demonstrated limited uptake so far, due to business, economic and technological barriers [37, 38, 48, 50]. Past ride-sharing studies focused mainly on ride-matching algorithms for ride-sharing optimization [2, 47, 63], dynamic ride-sharing pricing [2, 3], and the economic, social, transport, and environmental benefits of ride-sharing [19, 20, 83, 95, 111]. Studies on factors affecting ride-sharing use have been increased within the last decade (e.g., [11, 13, 14, 23]) showing the challenges and diversity of results per case study. A synthesis of information about factors that affect ride-sharing use and implementation barriers, is required to inform interested stakeholders and planners. To the best of our knowledge, there are no previous studies that review the user factors and barriers when implementing a ride-sharing service.

The aim of this systematic review is to understand, how successful ride-sharing services could be implemented and operated. This is achieved by recording and synthesizing data for online ride-sharing platforms, factors affecting users to ride-share (i.e., increase and decrease the likelihood to ride-share), and potential implementation barriers. The remainder of this paper is organized as follows: Sect. 2 outlines the methodological steps of this research and provides details for the publications that were collected and analyzed. Section 3 summarizes literature findings and results. More specifically, authors first review ride-sharing definitions and identify how the term is used in literature. Next, online ride-sharing platforms that were identified in literature are further explored in terms of operation status, starting year, location, and distance of service. User factors that are associated with the likelihood to ride-share are also recorded and presented. The third section synthesizes data from previous sections to discuss implementation barriers for ride-sharing services and make recommendations.

To provide a detailed understanding of ride-sharing it should be noted that users in this study are divided into drivers and passengers. Ride-sharing platforms refer to official providers or companies of ride-sharing services. Other topics, such as ride-sharing financial, economic or business models are not covered herein. Venues for further research are highlighted through the article.

## Methodology

This research focuses on a state-of-the-art analysis of ride-sharing that constitutes the basis for understanding different aspects, including online platforms and user factors and discusses potential barriers that prevent the successful implementation of ride-sharing systems. To achieve its purpose, the methodological approach builds on the principles of systematic literature review. A systematic review method helps researchers to develop a high-level overview of knowledge on a particular research area [22, 27, 56]. A systematic review means adopting a replicable, scientific and transparent process, in other words a detailed process that minimizes bias, through exhaustive literature searches of published and unpublished studies and by providing an audit trail of the reviewers’ decisions, procedures and conclusions [27].

**Fig. 1:**
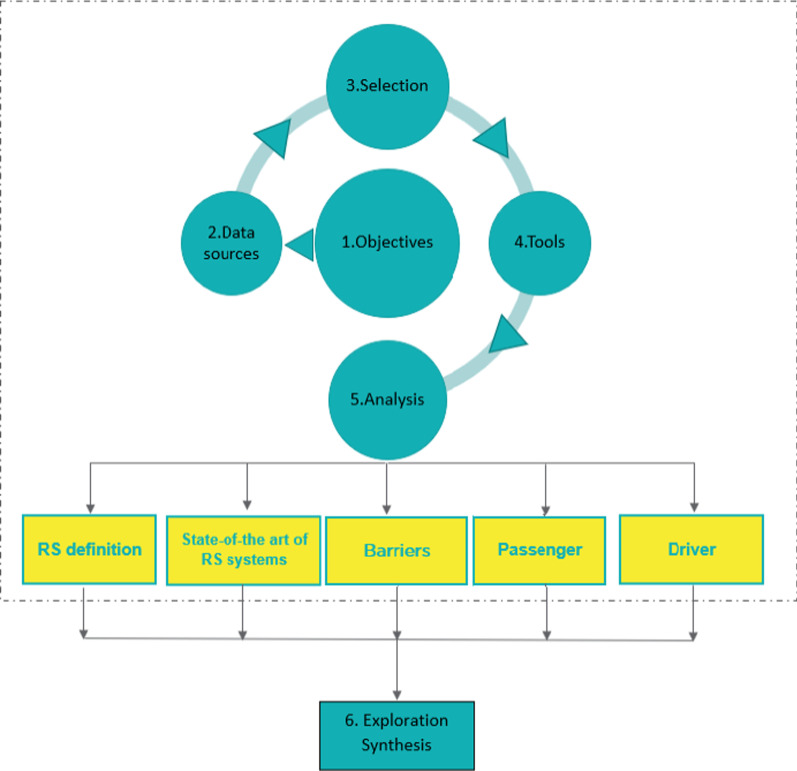
Methodological structure

The methodology focuses on the content of the publications, the research per se, rather than on their metrics. Although, more information regarding local ride-sharing systems may exist in different languages, we have limited the scope of this study to English-speaking publications, and we focus only on papers published in academic journals and conference proceedings, excluding books, chapters of books, thesis and dissertations. Following Moustaghfir [69], the methodological approach adopted, comprises of six parts (Fig. 1), as follows:

### Identification of objectives

Adapting the paper’s goal and the steps for performing a systematic literature review, the research questions (RQ) are shaped before starting to perform the review [27]. These are:

- RQ1: Does a universal definition for “ride-sharing” exist in literature, and how is ride-sharing defined?
- RQ2: Do ride-sharing online platforms (i.e., in operation and inactive) share common attributes?
- RQ3: What factors affect passenger and drivers to use ride-sharing?
- RQ4: What prevents ride-sharing systems from being successful?

Based on these four questions—four main objectives were identified as of high relevance to the understanding of ride-sharing services:

- Definition of a ride-sharing;
- State-of-the-art analysis of ride-sharing online platforms;
- Identification of factors affecting current and potential ride-sharing passenger and drivers.
- Synthesis and discussion of barriers for implementing a successful ride-sharing system.

### Identification of data sources and databases

The purpose of data collection is to collect the most representing research material and use the most recent information available. This step is composed of three sub-steps: Primary studies, search keywords, search database. Primary studies refer to the identification of relevant studies, to ensure first that the set research questions-objectives are valid, avoid duplication of previous work, and ensure that enough material is available to conduct the analysis. An initial search in “Google Scholars” and “science direct” by using the term “ridesharing” AND “review” resulted to three relevant studies, that review dynamic ride-sharing concept [2], ridesharing and matching criteria [38], and a meta-analysis exploring the factors that affect ride-sharing, which included 19 papers in the analysis [73]; however, none of them includes a review on ride-sharing platforms, user factors and barriers.

As a first step the keywords were identified to enable the conceptualization of the research and helped to target relevant articles. Prior selecting keywords, a shortlist of sharing mobility services was made. The keywords were defined by the authors based on their professional experience. Keywords related to shared mobility definition included: ride-sharing, carpooling, mobility as a service, MaaS, innovative mobility. Car-sharing publications, which refer to short-term auto use [20], were excluded from this research to focus exclusively on on-demand transport for passengers.

The terms “Ride-hailing” and “on-demand ride” were also excluded, as these two terms returned publications relevant to ride-sharing services that aim to financial gain (e.g., Uber, Lyft, etc.).

In literature, carpooling is a synonym for ride-sharing for non-profit reasons. The keywords ride-sharing and carpooling were constructed into search strings by using other keywords relative to the objectives, such as factors, users, passengers, barriers, constraints, legal-framework, drivers; resulting to strings: ride-sharing factors, ride-sharing users, etc. These search strings were used to conduct searches for all geographical areas. Factors that decrease the likelihood to ride-share and thus prevent ride-sharing implementation may be considered as barriers or constraints. Thus, authors included both terms as separate search terms for performing a complete review and synthesizing results. It should be noted that keywords ride-sharing and carpooling were typed in all possible formats, as these were found in literature: with a dash (–), with a space and as single words. We limited our research to articles published in English language within the last 30 years, from 1990 to 2020. Concurrently, authors and year of publication were also identified to perform a second search based on their names.

The data sources that were used to collect the necessary information and data include published journal and conference papers (Science Direct, Web of Science, Google Scholar, Wiley Online Library and Springer). Online platforms that were identified in these data sources, were further explored. The status and attributes of identified ride-sharing online platforms were not disclosed in the scientific manuscripts; therefore, a follow-up desk review conducted by focusing on online official websites and social-media of each provider.

### Selection of publications

The first task was to merge publications and exclude potential duplicates, thesis or dissertations, and publications that were not related to ride-sharing, such as publications focusing on taxi ride-sharing services. All duplicate publications were deleted; the remaining ones were exported to an excel file for screening. Definitions for different and partially overlapping concepts have emerged in publications’ titles, including ride-hailing (commercial, organized by companies), ride-sourcing and ride-pooling (commercial, organized by public institutions) [29, 35]. Publications not referring to ride-sharing or carpooling were eliminated by title screening. The second task was to identify if these publications refer to ride-sharing, carpooling or ride-hailing. This was achieved by reviewing each publication’s abstract. Abstract reviewing was performed by authors who are transportation experts. In some cases, the ride-sharing definition that was used in the study was not clear and authors had to review the introduction or/and the methodology of each publication (i.e., text review).

Each publication was recorded according to title, authors, year of publication and location of the study, and then it was reviewed to record specific features (when available) and build the database. These features refered to: (a) Ride-sharing definition, (b) Ride-sharing platforms (i.e., specific ride-sharing online platforms by name), (c) User factors—referring to factors affecting users (i.e., passengers and drivers) to use ride-sharing services, and (d) Barriers—referring to potential barriers and constraints that are faced in the implementation of ride-sharing services.

### Development of tools for data collection

For facilitating the data collection process, a template was developed. The developed template aimed to collect and organize information relative to ride-sharing online platforms, which is provided on the websites and social media of ride-sharing companies or related services, according to the following characteristics:

- Name of company/ride-sharing platform
- Potential barriers and provided incentives
- Country of operation
- Company/provider website
- Current status of ride-sharing platform (in/not in operation)
- Period of operation of the ride-sharing platform
- Provision of urban/interurban transport services (i.e., urban trips here are considered within the same city; interurban include all other trip types).

### Analysis

Collected information is analyzed and used as input to support each of the four objectives. Data are tabulated when possible, to support the objectives and are presented in the following sections.

**Fig. 2:**
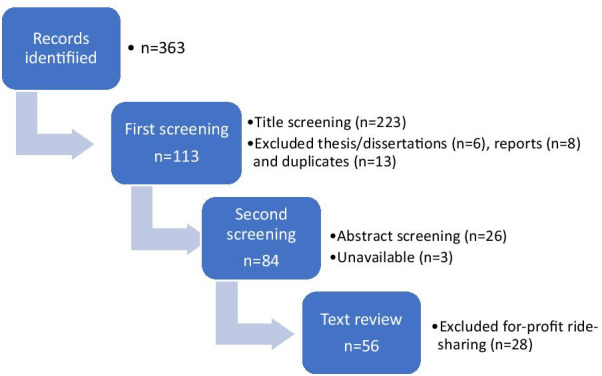
Number of publications in the review process

Figure 2 provides the flow diagram of publications included in the review [67]. The initial combined total number of publications was 363 articles. Following the first screening, 113 publications remained. The second screening identified if these publications refer to ride-sharing, carpooling or ride-hailing by reviewing their abstracts. Three articles that fulfilled the criteria, were not available in a database and thus were eliminated. Following the second screening, 84 publications remained. Following the text review, twenty-eight publications were found to use the term ride-sharing while referring to for-profit ride-sharing services such as Uber and Lyft (i.e., ride-hailing). Finally, 56 articles met the inclusion criteria for our review.

The majority of them use the term ride-sharing (n = 32) and carpooling (n = 23). It should be noted that one publication uses both the term ride-sharing and ride-hailing. Almost half of the studies were conducted in the US (n = 25) and one-quarter in EU and the UK (n = 19), with the rest being global (n = 2), in China (n = 4), in Canada (n = 3), in Australia, in New Zealand and in Asia (all n = 1). The majority of the studies focus on user factors (n = 32), while 15 of them discuss barriers related to planning and implementation of ride-sharing, and 18 mention at least one ride-sharing online platform.

### Exploration and synthesis

For each of the four objectives a discussion and synthesis of information is provided in respective sections, as outlined in the introduction.

## Results

The results of the literature review are summarized in Table 1.

**Table 1:** Summary of ride-sharing publications

Literature	Year	Location	Platforms	User factors	Barriers
Abrahamse and Keall [1]	2012	N. Zealand	●	●	
Agatz et al. [3]	2012	US	●		
Amey et al. [4]	2011	US		●	●
Bicocchi and Mamei [10]	2014	Italy	●		
Brownstone and Golob [11]	1992	US		●	
Buliung et al. [12]	2009	Canada	●		
Buliung et al. [13]	2010	US	●	●	
Bulteau et al. [14]	2019	France		●	
Chan and Shaheen [20]	2012	US			
Chaube et al. [23]	2010	US		●	
Ciari [24]	2012	Switzerland		●	
Ciari and Axhausen [25]	2012	Switzerland		●	
Correia and Viegas [28]	2016	Lisbon		●	●
Deakin et al. [30]	2010	US		●	
Delhomme and Gheorghiu [31]	2014	France		●	
Dorner and Berger [33]	2016	Germany		●	
Ferguson [37]	1995	US		●	●
Furuhata et al. [38]	2013	US	●		●
Gargiulo et al. [39]	2015	Italy	●	●	
Gheorghiu and Delhomme [42]	2018	France	●	●	
Guidotti et al. [45]	2017	Italy	●		
Gurumurthy and Kockelman [46]	2020	US		●	
Hartwig et al. [48]	2007	US			●
Heinrichs et al. [49]	2016	Germany		●	
Hwang and Giuliano [50]	1990	US			●
Javid et al. [52]	2017	Pakistan		●	
Jiang et al. [53]	2018	China	●		
Kelly [54]	2007	US			●
Kladeftiras and Antoniou [55]	2015	Greece		●	
Lee and Savelsbergh [57]	2015	US			
Lee et al. [58]	2016	US		●	
Li et al. [61]	2007	US	●	●	
Monchambert [65]	2017	France	●	●	
Morency [66]	2012	US		●	
Mote and Whitestone [68]	2010	US			●
Neoh et al. [73]	2017	UK		●	●
Nikitas et al. [74]	2017	UK	●		
Nourinejad and Roorda [71]	2016	Canada			
Olsson et al. [75]	2019	Global		●	●
Payyanadan and Lee [76]	2017	US			●
Shaheen and Cohen [82]	2019	US	●		
Shaheen et al. [83]	2012	US	●		
Shaheen et al. [84]	2017	France	●	●	
Stiglic et al. [90]	2016	US	●		
Tahmasseby et al. [92]	2016	Canada		●	
Tavory et al. [93]	2019	Global			●
Vanoutrive et al. [97]	2016	Belgium			●
Wang [100]	2011	China		●	
Wang et al. [102]	2017	Australia			●
Wang and Chen [101]	2019	US		●	
Wang et al. [103]	2018	US			●
Wang et al. [104]	2019a	China		●	
Wang et al. [105]	2019b	China		●	
Wilkowska et al. [107]	2014	Germany		●	
Xu et al. [108]	2015	US	●		
Yin et al. [109]	2017	France	●		

### Ride-sharing definition

Table 2 presents a sample of recent publications and ride-sharing definitions. A universally accepted definition for “ride-sharing” does not exist and the term “ride-sharing” is defined based on the context of each study.

**Table 2:** Ride-sharing definitions within literature

Literature	Year	Location	Definition
Abrahamse and Keall [1]	2012	N. Zealand	Carpooling is defined as the shared use of a private vehicle by the driver and one or more passengers (replacing the use of one or more other vehicles), generally for the purpose of commuting to and from work
Agatz et al. [2]	2011	US	Ride-sharing refers to a system where an automated process employed by a ride-share provider matches up drivers and riders on very short notice, which can range from a few minutes to a few hours before departure time
Brownstone and Golob [11]	1992	US	Carpooling (hereafter called ride-sharing) is defined in the Southern California sense as two or more occupants per vehicle
Chan and Shaheen [20]	2012	US	Ride-sharing is the grouping of travellers into common trips by car or van. When a ride-sharing payment is collected, it partially covers the driver’s cost. It is not intended to result in a financial gain. Moreover, the driver has a common origin and/or destination with the passengers
Furuhata et al. [38]	2013	US	Ride-sharing refers to a mode of transportation in which individual travellers share a vehicle for a trip and split travel costs such as gas, toll, and parking fees with others that have similar itineraries and time schedules. Ride-sharing is a system that can combine the flexibility and speed of private cars with the reduced cost of fixed-line systems, at the expense of convenience
Gargiulo et al. [39]	2015	EU	Ride-sharing is the transportation of persons in a motor vehicle when such transportation is incidental to the principal purpose of the driver, which is to reach a destination and not to transport persons for profit
Guidotti et al. [45]	2017	EU	Carpooling is the act where two or more travellers share the same car for a common trip
Kladeftiras and Antoniou [55]	2015	EU (Greece)	Dynamic ride-sharing and traditional carpooling both involve pre-arrangements, but dynamic ride-sharing differs in the fact that the scheduling of the trip occurs in a case-by-case basis
Lee and Savelsbergh [57]	2015	US	Dynamic ride-sharing is a recent alternative in which people with similar travel plans are matched and travel together. Ride-sharing systems, where participants with similar travel itineraries are paired together
Nourinejad and Roorda [71]	2016	Canada	Dynamic ride-sharing involves a service provider that matches potential drivers and passengers with similar itineraries allowing them to travel together and share the costs. These services are dynamic in nature since users announce their participation at any time by either requesting a ride as a passenger or offering a ride as a driver
Shaheen and Cohen [82]	2019	US	Shared ride services allow riders to share a ride to a common destination. They include ride-sharing (carpooling and vanpooling); ride-splitting (a pooled version of ride-sourcing/transportation network companies); taxi sharing; and micro transit
Wang, Winter and Ronald [102]	2017	Australia	Ride-sharing is a mode of transportation where a driver takes passengers on a non-commercial, e.g., shared cost basis, for accompanied costs such as petrol

Ride-sharing typically includes carpooling and vanpooling [20], while the term does not necessarily refer to consistent participation in the same ride-share service every day [20] neither to daily use of the service. Ride-sharing may be used by its passengers as a mode to complete their whole trip (i.e., origin to destination) or to complement public transport, with the focus of further incorporating public transport in the multimodal transport chain. In the latter context, ride-sharing aims to facilitate access for the first/last mile to public transport services, to optimize multimodality and on-demand mobility, thus reducing single-occupant trips, and finally to develop smart urban/rural transport areas. A ride-sharing definition that may be used for non-profit ride-sharing services is proposed according to Code of Virginia US [26] that defines “Ride-sharing” as the transport of persons in a motor vehicle when such transportation is incidental to the principal purpose of the driver, which is to reach a destination and not to transport persons for profit.

### Ride-sharing platforms

In total 29 ride-sharing online platforms have been identified in the reviewed literature (Table 3). The platform recommends a ride fee and passengers decide to accept it or not; from the total fee the provider retain a fixed amount to cover the transaction cost. Although this is the most common practice, in very few occasions (only 2% of the cases), drivers may decide what to charge passengers after reviewing the platform’s recommendation and this occurs for interurban ride-sharing services.

**Table 3:** Summary of ride-sharing platforms

Name	Continent	Year	In operation	Service distance
Auto strade carpooling [6]	EU	2009–	Yes	Interurban
Autoincomune [5]	EU	2012–2017	No	Urban
Avacar [7]	EU	2011–2013	No	Urban/Interurban
BlaBlaCar [8]	Global	2006–	Yes	Interurban
Bring-me [9]	EU	2011–2014	No	Urban
Car2gether [15]	Global	2010–2011	No	Urban/Interurban
Carriva [16]	EU	2008–	No	Urban
Carticipate [17]	Global	2008–2012	No	Urban
Casual carpool [18]	US	1990–	Yes	Urban
DiDi Hitch [32]	Asia	2015–	Yes	Interurban
GoCarma [43]	US	2007–	Yes	Urban
Gomore.dk [44]	EU	2005–	Yes	Urban/Interurban
JoJob (Italy, Spain) [51]	EU	2014–	Yes	Urban
Liftshare [62]	EU	1998–	Yes	Urban
Motar (Central Europe) [67]	EU	2007–	Yes	Urban/Interurban
MyLifts (aka EuroLifts) [70]	EU	1997–	Yes	Urban/Interurban
PoolMyRide [78]	Asia	2013–	Yes	Urban/Interurban
Poparide (Canada and US) [79]	Global	2010–	Yes	Interurban
Ride joy [85]	US	2011–2013	No	Interurban
RideShark (Canada and US) [80]	Global	2002–	Yes	Urban/Interurban
Roadsharing [81]	EU	2008–	Yes	Urban/Interurban
sRide [87]	Asia	2014–	Yes	Urban/Interurban
TwoGo [96]	US	2011–	Yes	Urban/Interurban
Viaggiainsieme [98]	EU	2010–2016	No	Urban
Ville Fluide [99]	EU	2008–2015	No	Urban
Waze carpool [106]	Global	2018–	Yes	Urban
youTrip [110]	EU	2009–	Yes	Interurban
Zebigo [90]	US	2010–2013	No	Urban
Zimride [112]	US	2007–2015	No	Urban/Interurban

In terms of geographical coverage, ride-sharing platforms operate in US, EU, Asia, and Latin America. Ride-sharing platforms that provide services to more than one of these geographic areas are classified as global. The majority of the ride-sharing platforms were found to operate in EU (48%) with 27% of them being in Italy; a high share compared to the rest of the EU countries, showing the attempts to promote ride-sharing in Italy. US- and Asia-based platforms accounted for 20% and 10% of all platforms, respectively, while 20% operate globally. Although, this geographic classification refers to countries or continents, rarely one service covers the totality of a country as in most cases, services operate in a specific city or several close-by cities.

Urban and interurban platforms cover roughly 42% and 20% of all platforms, respectively, while ride-sharing platforms that cover both urban and interurban trips account for 38% of all. Urban trips here are considered within the same city; interurban include all other trip types. Often, ride-sharing platforms that provide only interurban services provide booking access through a website platform, whereas access through a mobile application is not available. To our understanding this occurs because interurban ride-sharing platforms require low maintenance in terms of administration and matching algorithms. In these cases, drivers publish their trip in advance and passengers review trip details (i.e., trip cost, destination, time of departure, driver profile) and decide to join or not. Therefore, to avoid extra maintenance costs for the service, a mobile application is not available. Several ride-sharing platforms have ceased operations due to low demand; some of them have re-started operation under a different name or/and follow a different business model. Approximately, 62% of the surveyed ride-sharing platforms are currently in operation, whereas 38% have ceased their operation. The vast majority of ride-sharing platforms (93%) have started their operation in 2005 or after, while 62% were found to start operations in or after 2010, which might be explained by the rapid development of mobile applications and spread of smartphones. Smartphone annual sales doubled between 2007 and 2010 (i.e., 122.32 vs. 296.65 million units), and increased by a factor of 4.2 between 2010 and 2014 (i.e., 296.65 vs. 969.72 million units), to reach 1540.66 million sold units in 2019 [89].

An important aspect, to address safety and security concerns and improve the overall level of services, is users’ feedback, as all of the ride-sharing platforms allow users to provide “feedback” either through the provided platform, through the application, or both. The feedback platform allows users to comment and evaluate the seriousness and reliability of drivers and vice versa. To further increased sense of safety, some platforms provide the option to women to travel only with other women as co-passengers or even drivers (i.e., Avacar).

The procedure to access ride-sharing is the same in all cases: users enter the platform, register and then search for offered trips. Trips can be organized last-minute, however, some platforms (18%) offer the opportunity to pre-plan trips one to two days in advance (e.g., for interurban trips).

The matching mechanisms for 90% of the platforms are destination-based. Drivers, who offer a ride, insert the departure and arrival locations and wait for those looking for the ride to that destination or a location along the way. The passenger consults a list of available to find the one that best meets their needs (i.e., departure, arrival, time, crew members, etc.). Once the passenger selects the path of their interest, they may undertake the necessary agreements (e.g., meeting point, how to recognize themself, etc.). Ride-sharing platforms do not use a sophisticated algorithm with multiple criteria to find the perfect ride-match, opposed to ride-hailing platforms that incorporate more travel and user criteria [64]. Only one platform (i.e., TwoGo) was found to use an intelligent technology to analyze rides from all users to find the best fit for each user, and factor in real-time traffic data to calculate precise routes and arrival times.

Several incentives are used to promote ride-sharing, such as toll cost reduction [6], High Occupancy Vehicle (HOV) lanes in US [18, 43], free or discounted parking access in public or private areas [51, 88], public transport ticket discounts and collection of points that may be redeemed in companies that collaborate with ride-sharing services [8, 51]. For example, Autostrade [6] carpooling with at least 4 passengers pays 0.50 euros toll, instead of 1.70 euros, from Monday to Friday; or GoCarma [43] that uses Bluetooth to automatically detect if there are at least 2 people in the car so as to qualify for an HOV toll discount.

### User factors

Several studies in the literature focused on the exploration of users’ factors when using ride-sharing services (Table 1). User factors may be associated in a positive or negative way with ride-sharing. In the latter case they may also be considered as barriers to ride-sharing implementation. The literature shows that the strongest identified barriers for ride-sharing users are mainly psychological [1, 52, 91] with the most common ones being personal security, comfort and privacy [1, 52, 91]. This section summarizes these findings and identifies the factors that are associated with the likelihood of ride-sharing for passengers and drivers. The following subsections summarize factors and results for ride-sharing passengers and drivers, and Table 4 summarizes the studies and factors that are associated with the likelihood of ride-sharing.

**Table 4:** Summary of user factors associated with the likelihood to ride-share

Literature	Year	Location	Sociodemographic							Location		
			Marital status	Trip purpose	Income	Gender	Educational level	Age	Sustainability concerns	Travel distance/time	Lack of public transport/frequency	Area density
Abrahamse and Keall [1]	2012	N. Zealand								●	●	
Amey et al. [4]	2011	US			●					●	●	●
Brownstone and Golob [11]	1992	US				●				●		
Buliung et al. [13]	2010	US				●		●	●	●		●
Bulteau et al. [14]	2019	France			●					●		
Chaube et al. [23]	2010	US								●		
Ciari and Axhausen [24]	2012	Switzerland		●	●					●		
Correia and Viegas [28]	2016	Lisbon	●	●	●		●	●				
Deakin et al. [30]	2010	US										●
Delhomme and Gheorghiu [31]	2014	France		●		●			●	●		●
Dorner and Berger [33]	2016	Germany							●		●	●
Ferguson [37]	1997	US			●		●	●				●
Gargiulo et al. [39]	2015	Italy										
Gheorghiu and Delhomme [42]	2018	France						●	●	●	●	
Gurumurthy and Kockelman [46]	2020	US			●		●	●		●		●
Heinrichs et al. [49]	2016	Germany				●					●	●
Javid et al. [52]	2017	Pakistan	●						●	●		
Kladeftiras and Antoniou [55]	2015	Greece										
Lee et al. [58]	2016	US		●		●	●	●		●	●	●
Li et al. [61]	2007	US							●	●	●	
Monchambert [65]	2017	France		●	●	●		●		●		
Morency [66]	2012	US										
Neoh et al. [73]	2017	UK			●	●	●	●	●	●	●	●
Olsson et al. [75]	2019	Global				●			●	●		●
Shaheen and Cohen [82]	2019	US	●	●	●					●		
Shaheen et al. [84]	2017	France		●	●		●	●			●	
Tahmasseby et al. [92]	2016	Canada	●		●			●	●	●	●	
Wang [100]	2011	China			●				●	●		
Wang and Chen [101]	2019	US								●		●
Wang et al. [104]	2019a	China										
Wang et al. [105]	2019b	China	●	●	●			●		●	●	
Wilkowska et al. [107]	2014	Germany				●		●	●			

Literature	Year	Location	System					
			Trip cost	Security /trust	Incentives*	Matching information/availability	Lack of flexibility	Socializing
Abrahamse and Keall [1]	2012	N. Zealand	●			●	●	●
Amey et al. [4]	2011	US						
Brownstone and Golob [11]	1992	US			●			
Buliung et al. [13]	2010	US		●	●	●	●	
Bulteau et al. [14]	2019	France	●					
Chaube et al. [23]	2010	US	●	●	●	●		
Ciari and Axhausen [24]	2012	Switzerland						
Correia and Viegas [28]	2016	Lisbon		●	●		●	
Deakin et al. [30]	2010	US			●	●		
Delhomme and Gheorghiu [31]	2014	France	●				●	
Dorner and Berger [33]	2016	Germany		●			●	
Ferguson [37]	1997	US						
Gargiulo et al. [39]	2015	Italy	●	●		●		●
Gheorghiu and Delhomme [42]	2018	France	●	●				
Gurumurthy and Kockelman [46]	2020	US						
Heinrichs et al. [49]	2016	Germany		●		●		
Javid et al. [52]	2017	Pakistan	●	●	●		●	●
Kladeftiras and Antoniou [55]	2015	Greece	●	●				
Lee et al. [58]	2016	US	●	●			●	
Li et al. [61]	2007	US	●		●	●		●
Monchambert [65]	2017	France						
Morency [66]	2012	US				●		
Neoh et al. [73]	2017	UK	●	●	●	●		
Olsson et al. [75]	2019	Global	●	●	●			●
Shaheen and Cohen [82]	2019	US	●		●			
Shaheen et al. [84]	2017	France						
Tahmasseby et al. [92]	2016	Canada		●	●	●	●	
Wang [100]	2011	China						
Wang and Chen [101]	2019	US	●					
Wang et al. [104]	2019a	China	●	●				
Wang et al. [105]	2019b	China	●		●			
Wilkowska et al. [107]	2014	Germany	●	●			●	

^*^Incentives: Free parking, use of HOV lanes, ride-sharing services available in a company or University

#### Ride-sharing passengers

Ride-sharing research on passengers’ behavior tend to refer to identical factors, which can be grouped in various ways; for example, Buliung et al. [13] classified ride-sharing factors as socio-demographic, spatial, temporal, automobile availability, and attitudinal, whereas Neoh et al. [73] grouped them into internal (i.e., individual characteristics and reasons to ride-share) and external (i.e., policy measures to facilitate ride-sharing, location-based factors). Our study adapts Neoh et al. [73] approach with some minor adjustments, and groups factors into sociodemographic, location and system factors. Sociodemographic factors are factors associated with the passenger’s demographic and socioeconomic status, and beliefs such as environmental concerns; location factors refer to spatial characteristics of travelling, such as trip distance and time, and area density. System factors refer to the ride-sharing service environment, such as policies and incentives; system factors may be adjusted by the ride-sharing service provider. The factors per study that are reported in Table 4 were found to be statistically significant.

Several studies (e.g., [13, 14]) concluded that socio-demographic characteristics, such as marital status, gender, age and educational level are not significant; whereas behavioral factors are. Other studies, however, concluded that some socio-demographic characteristics, such as age, income and age, are associated to ride-sharing [28]. Females, younger workers, and those who live with others were found to be more likely to ride-share [58, 73]. Delhomme and Gheorghiu [31] found that women are almost three times more likely to use ride-sharing compared to men, while Lee [58] concluded that females who are younger than 55 years old are more likely to ride-share than older males. However, Ciari and Axhausen [25] concluded that female individuals in Switzerland are less attracted to ride-sharing, maybe for security concerns.

Education level was not a significant factor in the majority of the studies, while just a few found that education is related to ride-sharing, and more specifically, users that do not hold a degree are more likely to ride-share [58]. In terms of marital status, passengers between the ages of 25 and 34 were more likely to make commute trips (96%) versus non-commute trips (80%) by using ride-sharing services, and they were more likely to be single or married without children [92]. Specifically, a propensity towards ride-sharing is demonstrated among unmarried and divorced commuters.

The user or household income was not associated with increased likelihood to ride-share for the majority of the studies. Monchambert [65] used discrete mixed logit models to estimate the probability of mode choice and found that the ride-share value of travel time correlates with socio-economic variables. In other words, wealthier individuals seem to be willing to pay more to save travel time. Also, Ciari and Axhausen [25] concluded that persons with higher income and shorter trips tend to have a higher value of travel time savings, and thus, prefer ride-sharing compared to car, suggesting that it is also preferred to the other available modes.

Recent data, however, from the National Household Travel Survey in the US [72] indicated that ride-sharing passengers that have generally lower incomes, and minorities (typically Hispanics and African Americans) tend to ride-share more than other racial and ethnic groups [83]. Similarly, other studies concluded that lower income passengers are more likely to ride-share [14] or that ride-sharing maintains mobility for low-income passengers [4]. Ferguson [37] found that income has only an indirect impact on the choice to ride-share in lower income households, as income influences auto ownership and use. Higher vehicle ownership does not favor the utilization of ride-sharing services [37]; though, a study in China showed that the ride-sharing adoption rate was similar between households with cars and those without [100].

A strong relation was found between having ride-sharers among family/friends and colleagues, and engaging in ride-sharing [14, 33]. The tendency to adopt ride-sharing services is higher for multi-person households and households having more licensed drivers than vehicles [58]. The presence of children, elderly persons, or both, in the household is likely to have a negative effect on the adoption and frequency of use.

Findings on sociodemographic factors show that while these may be limited in their effect, when combined with system factors they may reveal a more stable status. As Olsson et al. [75] stated, other factors become more important for mode choice and are the focus of transport research.

In terms of trip characteristics, commuters who travel longer distances were found to be more willing to use ride-sharing services [58]. However, the in-vehicle time for public transport services was found to have a marginal impact on passengers’ propensity toward ride-sharing [64]. Based on transport mode shares for US, Australia, UK and Canada, there is some evidence that in the absence of adequate public transport services, commuters opt for ride-sharing [11, 33, 42, 58, 61, 104]. The purpose of the trip also plays a role, as ride-sharing is more likely to be used for work trips [24, 61] and for persons that have a full working or studying day. People who work full time and with flexible schedules are more likely than other workers and non-workers to adopt and frequently use ride-sharing.

Travel cost and travel time are associated with ride-sharing and are two of the main reasons for participating in ride-sharing services [14, 20, 61, 73, 105]. Commuters who travel short distances of a mile or two are less interested in dynamic ride-sharing than those who travel further because for short distances, the time required to arrange a ride is excessive [30]. For student passengers the desire to save on gasoline costs, followed by a preference to do other things during travelling, the reduced stress and travel time savings, increase the likelihood to ride-share [92].

Although, density employment centers in suburban areas were found to benefit public transit and nonmotorized modes more than ride-sharing [37], building and population density seem to increase the likelihood of ride-sharing [31, 58, 73].

Using microsimulation, Dubernet et al. [34] found that behavioral factors are the most limiting factor of ride-sharing; behavioral barriers, attitudes and perceptions were found to affect more the decision to use ride-sharing services than socio-demographics [97]. Research showed that enjoying travel with others, environmental considerations [31, 42] and socializing [39] affect at a significant level the choice to use ride-sharing services [61]. Other important factors for ride-sharing include security and trust [28, 48].

Several incentives have been provided occasionally to ride-sharing passengers, including reward programs that may provide money or gift cards for ride-sharing, access to green zones, (i.e., commuter rewards programmes that may provide money or gift cards for ride-sharing), etc. Such incentives showed that may attract ride-sharing participants from either single occupancy vehicles and/or public transit [28, 75, 82].

Although, the most prevailing results are summarized in this section, the literature review showed that factors affecting travellers to use ride-sharing services in some cases may differ among studies. For example, “income” is associated negatively [4, 13, 14, 82, 91] and positively [73, 103] with ride-sharing; “education” is associated negatively [58] and positively [73]; and “age” is associated negatively [58, 73] and positively [91]. Similarly, the location factor “area density” is associated negatively [4] and positively [31, 58, 73] with ride-sharing. Readers are strongly recommended to follow-up the study they are interested in, since different methods and statistics may have been used; thus, resulting to different factor results (i.e., not statistically significant) for specific cases.

#### Ride-sharing drivers

Ride-sharing users can offer a ride as a driver or request transport as a passenger. Drivers provide ride-sharing services and thus they are considered independent private entities. This approach is different from most traditional forms of passenger transport, where an authority or company owns vehicles and/or employs drivers. If the driver and the passenger agree on the proposed arrangement, the driver picks up the passenger at the agreed time and location.

Several surveys have been conducted to study the passenger’s behavior, however, few of these focused on the driver’s behavior. Respondents with a preference for driving only accounted nearly for 50% [13]. Approximately, 33% of the respondents stated that they would rather not offer a ride in the evening (18:00–24:00), while more than 52% of passengers stated that they would not accept a ride in the evening (18:00–24:00) [28]. Drivers indicated that departure time flexibility is the primary reason for driving instead of riding, as the highest share of them (74%) agrees that reducing flexibility is among reasons not offering a ride [33]. It is worth mentioning that other studies concluded that younger and older people tend to be passengers, while middle-aged people tend to be drivers [92]. Drivers appear to avoid ride-sharing as passengers as they feel anxious and stressed (usually studied as ‘locus of control’) when delegating the driving task to others [73, 97].

For drivers, a passenger’s profile is an important factor. Passengers, whose social network profile appears unattractive, incomplete or has low rating, have a lower chance of finding a ride offer [92]. Therefore, it becomes essential for potential passengers to have a trustworthy profile, including a picture, profile details, and contact information on a social network (e.g., LinkedIn, Facebook or Ride-sharing application). Similarly, the driver’s profile plays the most significant role in one’s decision to accept an offered ride [91]. This challenge has been largely addressed through the development of increasingly sophisticated ride-matching platforms. Another factor that differs between passengers and drivers is the payment method. Drivers prefer to receive the reimbursement in cash but passengers prefer to pay through a mobile payment platform, revealing drivers’ concerns over the certainty of the reimbursement [39].

## Discussion

Following the results of ride-sharing definitions, online platforms and user factors, this section synthesizes findings with barriers identified in literature (Table 1). Factors that prevent the successful implementation of ride-sharing services are grouped into economic, business, technological, behavioral and regulatory, to stimulate a discussion for implementing successful ride-sharing services.

### Economic barriers

Cost and convenience are important factors associated with the intention to start ride-sharing [1]. Time costs include the time that is required to set up an account in the ride-sharing application/website, the time it takes to find and book a ride through the application and the waiting time to join a ride. Booking time be insignificant when interurban rides are arranged but for daily rides this cost may seem significant to potential users [1]. Booking trips in advance is not convenient and may not suit to users that prefer instant arrangements and flexibility in their schedule [48]. Similarly, ride-sharing drivers are unwilling to experience more than 5–10 min delay in order to pick-up and drop-off passengers [64], suggesting time delay is a significant factor for joining a ride-sharing service as a driver. Ride-sharing platforms should try to minimize the time that it takes for different users to register, book and wait for a ride. Different users (e.g., based on trip purpose) show different sensitivity to waiting time, and the time range that each user may accepts should be investigated. The outcome of such research should be incorporated in the matching algorithm of the ride-sharing platform to address the needs for each user group. In this way it will be more likely these users to use more often ride-sharing services.

Also, fuel prices and fuel efficiency improvements for internal combustion engine vehicles seem to affect ride-sharing; in 1990s the decline in oil prices matched the decline in ride-sharing [37] from 20 to 13% [20]. Personal travel is less sensitive to gasoline price fluctuations than vehicular travel is, due to the ready availability of empty seats, which means that increased fuel prices will likely reduce vehicles on the roads, but not passenger travel. As fuel prices are not expected to decrease significantly in the short term and vehicle fuel efficiency improves in the meantime, ride-sharing may offer personal travelling until a cheaper alternative fuel replaces internal combustion engine vehicles [48].

### Business barriers

Ride-sharing platforms may integrate different business models to generate revenue. The two most used models are a commission fee based on the overall ride cost or a flat rate fee. The third alternative does not integrate any direct fee, and may rely solely on revenues from advertisements on the platform. In our data, only 7% of the platforms appear to charge a direct fee by either way [8, 91]. This implies that 26 platforms are neither set up as enterprises that aim to be economically sustainable in the future, nor they focus on growing their user base, thus they do not currently generate any profit. The level of success of these practices is questionable as several ride-sharing platforms stopped operating as outlined in Sect. 3.2 or they were transformed to ride-hailing services (e.g., Zimride became Lyft).

A solution proposed by Olsson et al. [75] to integrate ride-sharing platforms into the Mobility as a Service (MaaS) concept, where users shift from privately owned vehicles to monthly subscriptions for mobility services. Another recommendation is to integrate ride-sharing services with public transport in locations, where access to public transport is limited or frequency is low. Research showed that in these locations the likelihood to use ride-sharing services increases [64, 102]. In this way ride-sharing services should be partially subsidized to transfer travellers to public transport hubs.

Kelly [54] proposed to add ride-sharing to the list of modalities (currently public transit or vanpools) that are eligible for tax benefits. In this case the largest source of funds should come from the Regional Transportation Boards and state and federal agencies (in the case of US) that have as their mandate the construction and operation of transport systems.

Business models should focus on the community goals (e.g., reduce single occupancy vehicles, provide last mile rides) and users’ needs for each location. More experimentation is needed for designing and testing different types of incentives for different travel activities (work and non-work) to customize solutions per case [64, 75]. Incentives and subsidies should take into consideration the ride-sharing impacts to avoid under-subsidizing public transport modes or modes that generate less emissions (i.e., bike and micromobility). Unwanted barriers to ride-sharing such as taxation and insurance issues should be regulated to provide trust and confidence to its users. Analogously, ride-sharing parking and park and ride facilities should be carefully planned since they may generate additional traffic [97].

### Technological barriers

Ride-sharing platforms are supported by a mobile application or/and website to match potential drivers with passengers. The level of sophistication of the matching algorithm affects the ride-sharing participation either for existing or potential users. Also, even if drivers and passengers can be successfully matched, little is known about each individual participant regarding their driving history, annoying habits to co-passengers while ride-sharing (e.g., eating, smoking), criminal record, etc. [1]. People are significantly less willing to share a ride with strangers than with direct or indirect friends [102, 103]. The majority of the ride-sharing platforms rely on the user’s feedback to provide a secure ride to their participants. Therefore, imprecise or imperfect information to participants may hinder significantly ride-sharing.

A solution to this barrier could be the development of a greater ride-sharing database with collaborating capabilities with other databases, that can aggregate user data to increase the probability of matching up a driver and a passenger. As such, the integration of users’ information with other criminal or identification databases is an important step towards encouraging greater ride-sharing participation. Other social networking platforms like Google and Facebook can be incorporated in the ride-sharing platform to add extra credibility, and enable them as platforms to match ride-share users [57]. People with active profiles on social networking websites are less affected by trust issues when it comes to sharing a ride with people they have never met [39].

However, there are several emerging ethical concerns in big data analytics applications in public transport systems and ethical frameworks are required to provide a careful balance of benefits and risks driven by disruptive technologies [21]. A range of ethical impacts are identified relative to the implementation of data-driven transport systems, that constitute barriers to the development of smart mobility. Including but not limited to: trust, surveillance, privacy (including transparency, consent and control), free will, personal data ownership, data-driven social discrimination and equity [59]. The massive amount of information collected about people, privacy and security are reported as the main concern [77]. Concerning transport network companies, such as Uber or Lyft, significant evidence of racial and gender discrimination was documented in various experiments [41]. Additionally, elderly, people with low education and/or physical or mental problems are facing difficulties adopting emerging technologies, and may be excluded from a data-driven transportation system [21]. A recent study [88] noted the importance of social equity in smart cities and the need to address elderly people needs across various dimensions, including transportation.

Additionally, the outdated algorithms that are used in traditional ride-sharing platforms make difficult any last-minute schedule changes that a user would like to make [38]. One of the main reasons that ride-sharing, has fallen off dramatically over the past decade, at least in the US, is largely due to the inflexible nature of pre-arranged ride-sharing [68]. The maturing of internet adoption and more sophisticated algorithms allow internet-based ride-sharing platforms to overcome problems with schedule inflexibility [73]. Correia et al. [28] proposed that for managing schedule variations, a ride-sharing platform can be set to manage both traditional stable groups and a dynamic ride matching service. Dynamic ride matching services have proved to be very ineffective when applied independently; their success, however, strongly depends on the participants’ willingness to share a ride with a possible stranger [28, 102].

Despite multiple algorithmic improvements for ride-sharing, including real-time en-route planning, the mainstream ride-sharing applications are almost all trip-based, with specified fixed origin/destination pairs and thus low flexibility for destination choices. Frequently cited barriers to ride-sharing formation and use include: rigid scheduling and lack of matches between drivers and travellers [49, 66]. A gap that can be bridged by advanced software and algorithms, to provide enhanced matching. A new ride-sharing algorithm, called collaborative activity-based ride-sharing to address the barriers of trust and flexibility in ride-sharing was proposed [103], to increase favorable rides without sacrificing more detour time, which potentially encourages public acceptance of ride-sharing.

Lastly, acknowledgment of users' preferences will help service providers to build customized services to meet their travelling and behavioral needs. For example, older adults may require more space for wheelchairs [58] or students for special equipment, such as cameras or drawing equipment. Future research should focus on the effectiveness of matching algorithms by integrating more travelling and personal criteria to transform ride-sharing into a safe and entertaining mode.

Other major barriers that can be faced by enhanced mobile applications, include lack of information [4], belief that “nobody is going my way” [92], and aversion to handle direct money transactions [30].

### Behavioral barriers

Behavioral barriers have found to affect more the decision to use ride-sharing services than socio-demographics [97]. Research showed that enjoying travel with others, environmental and social consideration, trust and security affect at a significant level the choice to use ride-sharing services [48, 61]. Participation in activities such as reading a book, texting, or surfing the internet on their smartphone during the commute may be another influential factor relating to ride-sharing demand [92].

Ride-sharing systems that fail to provide the conditions for secure travelling pose barriers to a successful implementation of a ride-sharing system. The feeling of unsecure travelling may grow either by not sharing user profiles, user matching not based on user criteria, or lack of mobile applications that enhance security, for example not sharing your location. Research showed that the more information shared by users (i.e., time and place of the ride and information on interests and preferences), the more likely a matched ride could occur [65]. Poor flexibility is associate negatively with ride-sharing [28] and is also the main reason against sharing rides as passenger, with 66% supporting this argument [33]. Lee [58] suggests that having work schedule flexibility is associated with those who are more likely to use a non-rideshare mode, and most likely to telecommute, than to rideshare.

Also, ride-sharing services are more likely to be successful when an organization, resembling small communities, such as a company or a university provides these services in its premises [92]. Commuting with colleagues is probable increasing the levels of security, and provides an opportunity for socializing by sharing common topics of discussion.

Sharing roles, as opposed to drive-only or travel-only, has shown to affect success of ride-sharing, and appears to be the preferred approach by users, as they look to acquire both the economic advantages of driving some of the time, and the perceived psychological/comfort benefit of being a passenger [60].

As mentioned, and presented, the literature offers mixed findings on the relationship between demographic, behavioral characteristics and ride-sharing. Some relationships might exist between ride-sharing, specific users and their characteristics. However, after a specific user group adopts ride-sharing services, the practice may vary greatly within the user group, hence more complex relationships may ultimately describe the interactions that lead to such decisions [13]. A further analysis, will be able to explore the user characteristics for specific locations and travel purposes, and reveal clusters of users having similar characteristics, behavior and needs, to customize ride-sharing services, and to target specific users.

### Regulatory barriers

The European Union transport policy aims to ensure the movement of people and goods throughout the EU by means of integrated networks using all modes of transport (road, rail, water and air). However, within the existing transport legislation a common directive, among EU countries, for ride-sharing is not shared [36]. To best understand the ride-sharing, it becomes essential to understand the regulatory environment in which the services operate. The majority of EU-Members do not define or regulate ride-sharing; however, only 5 out of the 28 countries (i.e., France, Germany, the Netherlands, Spain and Sweden) provide a ride-sharing definition for non-commercial reasons (i.e., use of a motor vehicle with a driver and one or more passengers as part of a journey; the driver performs the trip on their own account and no remuneration is involved except the costs for the driver). Similarly, in US and Canada ride-sharing is not regulated as it operates on a non-profit basis. Setting an adequate legislative framework for innovative transport solutions is a prerequisite for their successful integration and implementation in existing transport systems. For example, countries that failed to set such a legislative framework for ride-hailing services (e.g., Uber in Denmark and Bulgaria) or for electric-scooters (e.g., Hive in Greece) were forced to cease the operation of these companies.

### Exploring users’ perceptions to develop a ride-sharing system

Limited information exists on the trip purpose of ride-sharing users, compared to the exploration of factors for passengers. Only a few studies in the literature review focused on travelling for work or educational purposes (i.e., travel to campus/university), while leisure/recreation and shopping trips are usually not considered. Similarly, Wilkowska et al. [107] suggested that little analysis is performed on trip purposes other than work. Teal [94] identified three types of ride-share users based on how they ride-share: (1) Household (travel only with household members), (2) External (travel with unknown individuals), and (3) Passengers. Gheorghiu and Delhomme [42] identified ride-sharing trips for work, children (picking up and/or taking other children to school and for children’s leisure activities), leisure, and shopping. The same study concluded that the longest ride-sharing trips were attributed to work purposes, the shortest to shopping, while leisure and children-related trips had approximately the same reported average length. Vanoutrive et al. [97] investigated the influential factors for pre-organized ride-sharing and found that different travel purposes (e.g., to home versus to workplace) bounded with their corresponding travel directions, yielded different ride-sharing rates. Also, the spatial distribution of travel demands and social networks affected matching rates [103].

Aforementioned barriers show that an understanding of the users’ behavior has the potential to provide insights and result to customized user recommendations for developing a successful ride-sharing services. A grouping of ride-sharing users is suggested on the basis of trip purpose, based on literature findings as presented above. Four user types are considered to cover the majority of trip activities, thus the majority of users:

1. Household work user (Trip to work with at least one person from the same household),
2. Solo work user (Trip to work with unrelated individuals),
3. University and college user (Trip for educational purposes with or w/o unrelated individuals)
4. Entertainment/shopping user (Trip for recreation and entertainment purposes (shopping is included here) with or w/o unrelated individuals).

Work users are divided into household and solo driving as several studies have focused on ride-sharing and commuting to work [30, 42, 97], and recent data suggested that household ride-sharing likely represent the largest share of arrangements [66]. Solo drivers appear not to be so favorable about using ride-sharing services [1], thus, the research findings (i.e., increased work-based ride-sharing shares and low penetration upon solo drivers), stress the need to consider and study this user type separately in order to design and form customized initiatives to promote ride-sharing. Ride-sharing should be also considered for recreation/entertainment activities, since some of these activities are fixed in terms of time, day and place (e.g., grocery shopping, training)”. The user types apply to both passengers and drivers, as there is no evidence that role preferences (i.e., passenger or driver) are associated with specific trip purposes.

Finally, further research to accommodate the needs of passengers that may combine ride-sharing with public transport (i.e., bus, rail, metro) is required to explore and determine the factors that affect use of ride-sharing. Apart from factors discussed in earlier sections, other factors may be considered, such as travelling time when using ride-sharing with public transport, and travel preferences (e.g., seat preferences, accessibility needs) when travelling with public transport.

### Practical implications

Our review findings are used to summarize and propose practical recommendations to service providers to enhance the popularity of ride-sharing systems; thus, increase ride-sharing demand. Economic factors, including time, appear to affect the willingness of users to use ride-sharing systems. The time to register in a platform and the process to find and book a ride either instantly or in advance, and the economic benefits of using ride-sharing are dominant factors for potential users. Ride-sharing service providers should develop and release an easy-to-use mobile application to support their services, which will be linked to a web-based platform to provide access for all travellers complying with local accessibility regulations; in this way a one-time registration will be required. Pre-booking rides is also perceived inconvenient by some users [48], which prohibit them from ride-sharing. Real-time ride-sharing [2] which brings together travellers with similar itineraries and time schedules on short-notice should be considered and adopted. Minimization of drop-off/pick up locations through optimization of meeting points and routes is also proposed to relax time constraints for potential passengers that appear to be sensitive to time delay.

Although, the studied ride-sharing systems do not offer financial benefits for the driver and the passengers, incentives are essential towards attracting more users. The service provider through the application should provide various financial incentives to increase the number of people who are eager to provide ride-sharing services (i.e., drivers); such incentives may include booking of parking spots, parking discounts and/or free passes in parking lots. Additionally, ride-sharing incentive programs for passengers may be developed to integrate cash or/and reward incentives. Direct cash incentives may be offered by companies to their employees in exchange for their parking space at work, while public authorities may also provide short-term cash incentives to new ride-sharing users. Georgia’s Cash for Commuters program offered a $3 USD per day incentive per new user for 90-days to try ride-sharing. It was found that 57% continued to ride-share 18 to 21 months after the initial incentive period [86]. Awarding points for ride-sharing trips that may redeemed in collaborative green-businesses and public transport schemes will also attract more users and highlight the relationship between ride-sharing and sustainability.

Marketing and promotion of ride-sharing services and their benefits will likely introduce the concept of ride-sharing to new users. The mobile applications and platforms may highlight the benefits to environment when travelling with others, while also disclosing that this mobility solution complies with national regulations related to COVID-19 passenger restrictions. Mobile applications, in the trip booking page, should provide a comparison of carbon dioxide and cost savings between private vehicle and ride-sharing to provide instant comparisons.

Mobility by public transport, railway, airplanes and ferries has been characterized as of high-risk activity that enables COVID-19 transmission, due to limited space that users have to share. As a result, ridership in public transport systems has decreased, while use of private vehicles has increased [64]. However, the share of travellers before and after the first COVID-19 lockdown period remained approximately constant. Ride-sharing provides a transport alternative that has the potential to provide mobility in a safe and controlled environment, that public transport may not be capable of guarantying. For example, the mobile application may ask users to provide their vaccine certificate in order to use the service.

Enhancing security by using several methods should be a priority for all ride-sharing services, since it affects the willingness of users to ride-share [48, 61]. The option to users to share their location in real-time with their contacts or other ride-sharing users should be implemented in the mobile application. A rating system, for both passengers and drivers, should be developed to provide feedback for all ride-sharing users. Such a mechanism will allow users to judge whether to accept or decline the offered ride, based on their perception. In this way, users may feel in control of their ride, and enjoying a sense of security. A list of regulations to ensure a safe and secure ride should be also provided to potential travellers, including abusive language, physical contact, unsafe driving, etc. Finally, an alarm button in the application could be added to notify the service provider in case of emergency by recording and forwarding the location and travellers’ information at the time of the incident.

## Limitations and strengths

The present systematic literature review focused on ride-sharing online platforms, factors and barriers, and did not include impacts or ride-matching algorithms. While these aspects are equally significant to the design of a successful ride-sharing service, the present study was conducted by recognizing that: (a) studies in the field of optimization and matching algorithm should be studied separately to focus on programming and technology aspects, and (b) studies on impacts of innovative transport systems, such as for ride-sharing, are challenging since the methods and tools to perform exhaustive life cycle assessments are limited.

We performed an extensive literature review that included 56 publications, while for 32 of them the factors that affect ride-sharing were extracted. Our results may help ride-sharing providers and transport planners to design and implemented successful ride-sharing services. However, the study suffers from certain limitations. The exclusion of grey literature and project reports could have been a limiting factor, in that it is possible that significant new findings might have been overlooked related to ride-sharing services. However, it should be noted that official websites of identified ride-sharing platforms were reviewed to collect specific data per platform. Also, the small number of ride-sharing platforms that was identified might led to not sufficient interpretation of the situation. In this aspect the informal character of ride-sharing should be considered, which leads to platforms that are not recorded or are not possible to target them as they operate in local social media and languages. Similarly, exploring regulatory barriers per country is hindered by language restrictions; likely local governmental documents may contain more information. Aspects of automated vehicles in ride-sharing were not considered either, which is an emerging field of discussion. Whether automated vehicles will be used for ride-sharing, as privately owned cars or in the form of service by ride-hailing services (e.g., Uber or Lyft) remains unknown [75]. The vague definition of ride-sharing might has also limited our findings. We are aware that there exist other forms of ride-sharing such as vanpooling, hitchhiking or slugging, that have not been considered.

Acknowledging these limitations, we do believe that this review provides important insights about official online platforms, what barriers exist, and who is likely to ride-share. Considering these aspects, transportation planners could be assisted and guided when planning a ride-sharing service, and choose more wisely which parameters should be customized and what users should target for, to implement a successful ride-sharing service.

## Conclusion

The systematic literature review of ride-sharing studies allowed us to have a comprehensive overview of academic publications dealing with ride-sharing platforms, user factors and barriers. These publications were selected using keywords that refer to ride-sharing, carpooling, barriers and factors. The systematic and comprehensive approach in this review adds strength to the research of economic, technological, business, behavioral and regulatory barriers on ride-sharing operation and success. Improving ride-sharing online platforms and applications and providing more features to users to customize their ride will likely generate positive impacts for ride-sharing.

Findings from this study provide insights and aspire to provide a comprehensive understanding of barriers and factors in decision-making process about ride-sharing. These findings could have important implications for urban and transport planners and policy makers to implement tailored solutions to users’ needs and socio-demographic characteristics. The results can be used as input to transport planning, policy-making and ride-sharing providers: revealing the potential barriers, enabling user-centered design environment, and providing recommendations for a successful ride-sharing service.

It appears to be a norm for location and system factors that affect users’ willingness to ride-share, however in some cases mixed findings exist between socio-demographic factors and ride-sharing. A limitation in existing research is the time of the study or the absence of studies before and after implementing a ride-sharing service. After a specific user group adopts ride-sharing, the practice may vary greatly within this user group, resulting to more complex relationships [14]. An ex-post evaluation of new introduced ride-sharing services has the potential to study and capture these relationships.

Additionally, it becomes important to examine the factors related to solo driving in each society for all travel activities and design customized interventions to target the behavior of solo drivers. Initiatives that aim to encourage solo drivers to start ride-sharing, could address some of the perceptions around the comfort and the convenience of driving alone versus ride-sharing. Public transport, walking, and biking are strong alternatives for passengers that avoid travelling alone, reducing the potential market for ride-sharing. For this reason, the estimates of participation rates must be considered case-specific, and decision makers have to consider whether to open and market the service to all or to focus on solo drivers. Continuous collection of user feedback through the ride-sharing platforms, and periodic reports from ride-sharing users is an important aspect in developing and improving ride-sharing programs.

The provision of ride-sharing policy is a rather interesting and complicated task that should take into account local and regional characteristics (i.e., demographics, economy, users, geography, transport). Further research is required to evaluate the relationship that exist between users and ride-sharing for existing (i.e., revealed experience) and potential (i.e., stated preference) users. Future directions will be towards exploring the user factors related to specific user-activities and ride-sharing. Additional system factors (e.g., ride safety, information regarding the vehicle condition, feedback method, etc.) should be explored to assess their impact on using ride-sharing services, while the most significant ones should be further investigated (e.g., to explore ride safety in terms of user identification method, sharing the ride online and payment method, etc.) to provide customized criteria that may be implemented within ride-sharing algorithms to optimize user-matching and experience.

## Data Availability

The datasets generated and/or analyzed during the current study are partly publicly available due to contractual restrictions. These can be found in Deliverable 2.2. State-of-the-art of ride-sharing in target EU countries, Horizon EU funded project Ride2Rail.
